# Melanoma Screening with Cellular Phones

**DOI:** 10.1371/journal.pone.0000483

**Published:** 2007-05-30

**Authors:** Cesare Massone, Rainer Hofmann-Wellenhof, Verena Ahlgrimm-Siess, Gerald Gabler, Christoph Ebner, H. Peter Soyer

**Affiliations:** 1 Department of Dermatology, Medical University of Graz, Graz, Austria; 2 Department of IT and Telecommunications, Graz University Clinics and General Hospital, Graz, Austria; Memorial Sloan-Kettering Cancer Center, United States of America

## Abstract

**Background:**

Mobile teledermatology has recently been shown to be suitable for teledermatology despite limitations in image definition in preliminary studies. The unique aspect of mobile teledermatology is that this system represents a filtering or triage system, allowing a sensitive approach for the management of patients with emergent skin diseases.

**Methodology/Principal Findings:**

In this study we investigated the feasibility of teleconsultation using a new generation of cellular phones in pigmented skin lesions. 18 patients were selected consecutively in the Pigmented Skin Lesions Clinic of the Department of Dermatology, Medical University of Graz, Graz (Austria). Clinical and dermoscopic images were acquired using a Sony Ericsson with a built-in two-megapixel camera. Two teleconsultants reviewed the images on a specific web application (http://www.dermahandy.net/default.asp) where images had been uploaded in JPEG format. Compared to the face-to-face diagnoses, the two teleconsultants obtained a score of correct telediagnoses of 89% and of 91.5% reporting the clinical and dermoscopic images, respectively.

**Conclusions/Significance:**

The present work is the first study performing mobile teledermoscopy using cellular phones. Mobile teledermatology has the potential to become an easy applicable tool for everyone and a new approach for enhanced self-monitoring for skin cancer screening in the spirit of the eHealth program of the European Commission Information for Society and Media.

## Introduction

The development of user-friendly technology has brought personal digital assistants (PDA) and cellular phones into everyday use. The power of these devices allows their use in more demanding tasks such as processing medical images; their use in telemedicine and particular in teledermatology has been recently proven and the name “mobile teledermatology” has been coined.[1], [2] In the first pilot studies limitations in image definition of cellular phones have been found, because the optics of the first generation cellular phones did not allow close-up or macro imaging.[1]–[3] Nevertheless, these studies have shown the usability and the feasibility of these new devices in teledermatology.[1]–[3] In fact, the unique aspect of mobile teledermatology is that this system might become a filtering or triage system allowing a more sensible approach for the management of patients with emergent skin diseases.[1]–[5] In addition, mobile teledermatology may also become a powerful screening tool for malignant cutaneous tumors. Skin cancers constitute nowadays the most common malignancies in the Caucasian population and the incidence has reached epidemic proportions.[6] Non-melanoma skin cancer (basal cell carcinoma, BCC, and squamous cell carcinoma, SCC) have an estimated incidence of over 600.000 cases per year in the U.S. (with a ratio of BCC to SCC of 4∶1), 20 times greater than that of melanoma.[7] In the past 25 years melanoma incidence has increased most rapidly than any other cancer, being now 18 new cases per 100.000 population per year in U.S.[8] Melanoma survival is related to its stage depending directly on melanoma thickness. A non ulcerated melanoma thinner than 1 mm has a 5-year survival of 95%, while an ulcerated melanoma thicker than 4 mm and with node metastases has a 5-year survival of only 24%.[9] For this reason early detection of thin melanoma is crucial, as the surgical excision today is the only life-saving approach.[10], [11] The accuracy of traditional clinical diagnosis of melanoma ranges between 65–80%.[12] Moreover, the naked-eye examination based on the ABCD system may fail to detect the so called small melanomas as well as melanoma regular in shape and/or colour.[13] Dermoscopy (in the past also called epiluminescence microscopy, dermatoscopy, surface microscopy), a recent, non-invasive, *in vivo* technique, has the potential to improve up to 49% the diagnostic accuracy for melanoma if used by experts.[14], [15] In this study we investigated the feasibility to perform melanoma screening with both clinical and dermoscopic images acquired using a new generation of cellular phones.

## Materials and Methods

**Figure 1:**
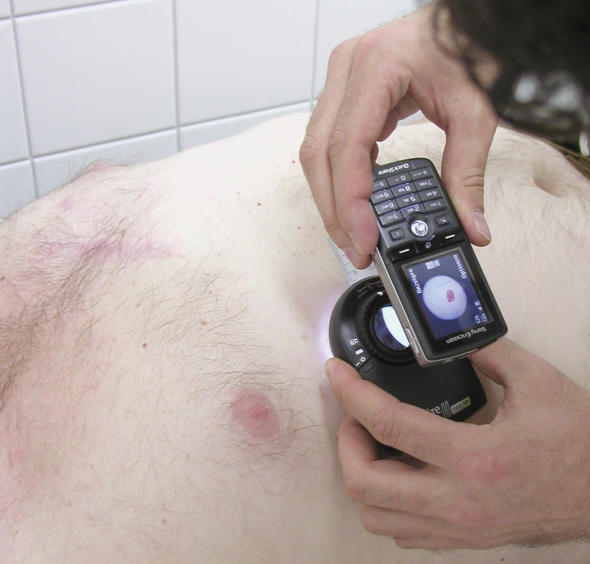
This dermoscopic image of a pigmented skin lesion has been captured applying the cellular phone on a pocket epiluminescence microscopy device.

**Figure 2:**
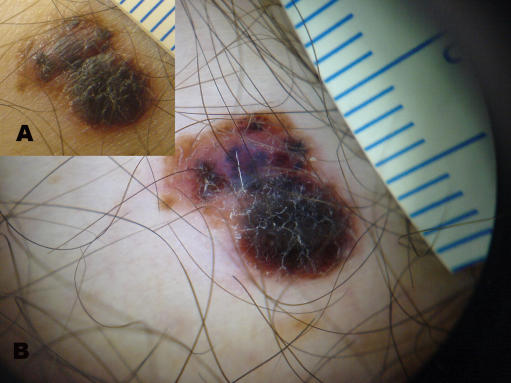
Clinical (A) and dermoscopic images (B) of a melanoma (case 9).

Eighteen consecutive patients (M:F = 12:6; mean age: 43,38; median age: 45; range: 14–78) were selected in the Pigmented Skin Lesions Clinic of the Department of Dermatology, Medical University of Graz, Graz (Austria) during two routine working days. Only patients who agreed to the study and signed the patient consent were enrolled. The face-to-face (FTF) diagnoses (16 benign lesions and 2 melanomas, Table 1) were made in each case by the same board-certified dermatologist (RHW). Images have been acquired under routine conditions and without additional light sources using a Sony Ericcson K 750i with a built-in 2 megapixel camera with autofocus, macro mode and zoom. In each case a close up clinical image and a dermoscopic image applying the cellular phone on a pocket dermoscopy device with a 25 mm 10× lens (DermLite II PRO HR (3Gen, LLC - Dana Point, USA); Figure 1) has been taken. Images had 1632×1224 pixels resolution with macro mode and were stored in JPEG format with an average size of 357 kilobytes (range 256–471 kilobytes). Images were transferred and saved on a computer using an USB port. Subsequently 2 images of each case (a clinical and a dermoscopic image) without clinical data were sent to 2 teleconsultants (HPS, CM) via a virtual private network (www.dermahandy.net/default.asp, e-derm-consult GmbH - Graz, Austria) based on store-and-forward systems (SAF; Figure 2).[16] The 2 teleconsultants reviewed the cases independently from each other and answered directly on the web application. A LAN connection was available for both teleconsultants. One of them used a Sony VAIO with a 15.4″ LCD screen; the other a Fujitsu Siemens Computer with a 15.4″ LCD screen. Both teleconsultants reviewed firstly the clinical images, made their clinical diagnosis and afterwards reviewed the dermoscopic images of each case and provided the dermoscopic diagnosis. Teleconsultants were asked to give the specific diagnosis for each case (i.e. “dysplastic nevus”, “blue nevus”) and only one diagnosis was accepted. The telediagnoses were compared with the FTF diagnosis which was taken as correct. Generic diagnoses as “nevus” have not been accepted and if more than one telediagnosis had been given, only the first one was considered as correct. We defined as diagnostic agreement the concordance between the telediagnosis and the FTF diagnosis. Excisions of the lesions with consequent histopathologic diagnosis were performed in 3 cases (2, 9 and 18). Teleconsultants were also asked to judge the quality of each image with the following scale: poor, fair, good, excellent (Table 1).

**Table 1:** FTF diagnosis and telediagnosis of teleconsultant A and B.

Case #	FTF diagnosis	Clinical telediagnosis		Dermoscopic telediagnosis		Image quality of clinical pictures		Image quality of dermoscopic pictures	
		A	B	A	B	A	B	A	B
1	blue nevus	1	1	1	1	Poor	Fair	Fair	Good
2	dysplastic nevus¶	1	1	1	1	Fair	Fair	Fair	Fair
3	dysplastic nevus	1	1	1	1	Poor	Poor	Fair	Fair
4	recurrent nevus	1	1	1	1	Fair	Fair	Fair	Fair
5	dysplastic nevus	1	1	1	1	Good	Good	Good	Good
6	dysplastic nevus	0*	1	0*	0*	Poor	Poor	Fair	Fair
7	seborrheic keratosis	1	1	1	1	Fair	Fair	Fair	Fair
8	angioma	1	1	1	1	Fair	Poor	Fair	Fair
9	melanoma¶	1	1	1	1	Excellent	Good	Excellent	Good
10	congenital nevus	1	1	1	1	Fair	Fair	Poor	Poor
11	dysplastic nevus	0†	1	1	1	Poor	Poor	Poor	Poor
12	dysplastic nevus	1	1	1	1	Poor	Poor	Fair	Fair
13	seborrheic keratosis	1	1	1	1	Excellent	Good	Excellent	Good
14	congenital nevus	1	1	0‡	1	Excellent	Excellent	Good	Good
15	acral nevus	1	1	1	1	Good	Good	Good	Good
16	dermatofibroma	1	0§	1	1	Good	Fair	Good	Good
17	angioma	1	1	1	1	Fair	Poor	Good	Good
18	melanoma¶	1	0||	1	1	Fair	Fair	Good	Good
**Diagnostic agreement**		16/18	16/18	16/18	17/18				
%		89%	89%	89%	94%				

FTF: face to face

A: teleconsultant A

B: teleconsultant B

1: agreement with FTF

0: disagreement with FTF

* clinical and dermoscopic telediagnosis: melanoma

† clinical telediagnosis: melanoma

‡ dermoscopic telediagnosis: melanoma

§ clinical telediagnosis: dysplastic nevus

|| clinical telediagnosis: seborrheic keratosis

¶ diagnosis confirmed histopathologically

## Results

Regarding the clinical images, the 2 teleconsultants agreed both with the FTF diagnosis in 89% (16/18); two dysplastic nevi (case 6 and 11) have been overdiagnosed as melanomas, a dermatofibroma (case 16) was diagnosed as dysplastic nevus and a melanoma (case 18) was underdiagnosed as seborrheic keratosis (Table 1). Reporting the dermoscopic images, the diagnostic agreement was 89% (16/18) in teleconsultant A and 94% (17/18) in teleconsultant B, respectively. A dysplastic nevus (case 6; both teleconsultants) and a congenital nevus (case 14; teleconsultant A) were overdiagnosed as melanomas (see also Table 1). The interobserver agreement among our 2 teleconsultants was of 89% and 94% for the clinical and dermoscopic telediagnoses, respectively. Quality of clinical images has been judged poor for 11 cases (31%), fair for 14 cases (39%), good for 7 cases (19%) and excellent for 4 cases (11%). Concerning the dermoscopic images, 4 cases (11%) have been judged poor, 15 cases (42%) fair, 15 cases (42%) good and 2 cases (5%) revealed excellent image quality.

## Discussion

Teledermoscopy represents a recent development of teledermatology. Dermoscopic images of pigmented skin lesions can be transmitted through internet to remote teleconsultants. The feasibility of teledermoscopy has been already proven by previous studies. In 1998, Provost et al. showed a high concordance in the diagnosis of atypical (dysplastic) melanocytic nevi and early melanoma between four different clinicians when comparing conventional slides with transmitted, compressed, digitized images.[17] One year later, in 1999, Piccolo et al. found a diagnostic concordance of 91% among FTF diagnosis and telediagnosis of 66 pigmented skin lesions sent via-email to a remote teleconsultant.[18] In 2000, in a subsequent multicentre study the same authors reported an average of correct telediagnoses of 85% in a subset of 43 cutaneous pigmented skin lesions sent by e-mail to 11 colleagues with different degrees of experience in dermoscopy.[19] In the same year, Braun et al. reported a teledermoscopic study in which six private dermatologists sent clinical and dermoscopic images of 55 pigmented skin lesions to the Department of Dermatology at the University of Geneva for teleconsultation over a period of 6 months. Their results showed that the diagnostic accuracy of teledermoscopy was superior to the one obtained on a FTF basis.[20] Recently, Moreno-Ramirez and colleagues evaluated teledermoscopy as a filtering system on 219 pigmented skin lesions. Teleconsultations were sent from general practitioner (GP) to the pigmented skin lesion clinic of the Department of Dermatology, University of Seville, in Seville/Spain. The outcome of the teleconsultation was that 49% of the patients were referred to the FTF clinic. The authors found an high agreement among the teleconsultants for both the diagnosis (κ = 0,91) and for the management options (κ = 0,92).[21] In particular, teledermoscopy seems to be suitable mostly as a triage system. In fact, Carli et al. stated that the examination of lesions (including dermoscopy) without contact with the patient is associated with improper management in about 30% of equivocal melanomas.[22]

The present work is the first study performing mobile teledermoscopy using cellular phones with an in-built camera. Moreover, this is the first time that a simply hand-held dermoscopy device has been used for a teledermoscopy study. In fact, we captured the dermoscopic images applying directly the cellular phone on a pocket dermoscope while in all the previously reported teledermoscopic studies the images had been acquired with an integrated digital dermoscopy device.[15] Again, in contrast with previous studies that transmitted the images via e-mail, we have tested a specific web application suited for teledermoscopy (www.dermahandy.net/default.asp). Comparing our results with those of previous studies in mobile teledermatology using cellular phones and PDA, it is not surprising that results are better when using the new generation of cellular phones.[1]–[3] In fact, the resolution of the in-built cameras in the new generation of cellular phones resolved the issue of the image quality found in the previous reports. This problem was due to limitations of the optics of the first generation cellular phones that were not designed for close-up macroimaging thus resulting in out of focus close-up images.[1]–[3] Still the images captured with the new generation of cellular phones are far from being perfect. In fact, 31% and 11% of clinical and dermoscopic images, respectively, have been judged by our teleconsultants as of poor quality as images revealed low sharpness and were not perfectly in focus. However, it seems likely that the routine conditions under which images have been captured were responsible for the low image quality rather than technical limitations. Although image quality did not represent an impediment to formulate the correct diagnosis in most instances, the reduced image quality in cases 6 and 11 might have influenced the telediagnosis. Further studies on larger series of cases are needed to study the influence of image quality on mobile telediagnoses.

Considering our clinical telediagnoses, a melanoma (case 18) has been underdiagnosed by one of our teleconsultants, 2 benign melanocytic nevi (cases 6 and 11) have been overdiagnosed as melanoma and a dermatofibroma (case 16) has been reported as a dysplastic nevus. Interestingly, reporting the dermoscopic images of case 11, 16 and 18 the teleconsultants changed their diagnoses achieving the agreement with the FTF and the 2 melanomas within the 18 pigmented skin lesions were correctly identified dermoscopically by both teleconsultants. These results may be explained by the fact that the clinical image alone in some cases is not enough to reach the correct diagnosis of a given melanocytic proliferation and underlines the value of dermoscopy in the diagnosis of melanoma.[11], [15] In case 6 the two teleconsultants agreed on the diagnosis of melanoma while the FTF diagnosis had been dysplastic nevus. It must been underlined that clinical data had not been provided to our teleconsultants in order to test their genuine capacity to formulate a telediagnoses. Case 6 was a dysplastic nevus from a 42-year old woman with a dysplastic nevus syndrome with clinically numerous atypical moles similar to the one that has been included in the study. Thus the lesion in debate was interpreted by the FTF dermatologist as not revealing the ugly duckling sign and just monitored and not excised.[23] Without this most important clinical information both teleconsultants overdiagnosed clinically and one also dermoscopically this lesion as melanoma.

Currently, in many medical specialities research in telemedicine is focusing on developing and testing new ways to utilize cellular phones for home-based health data acquisition. Home monitoring using information and communication technologies is particularly suitable for managing chronic diseases and a number of clinical trials have indicated the value of this concept to optimize therapy in hypertension, diabetes, asthma as well as to reduce hospitalization for patients with heart failures.[24] The feasibility study presented herein shows for the first time the potentiality of mobile teledermatology and mobile teledermoscopy as a triage system for pigmented skin lesions. In accordance with the new formulated concept of “person-centred health system” this approach could open up new horizons for persons with numerous moles and suspicious pigmented skin lesions.[25], [26] In fact, one of the cardinal points of the eHealth program of the European Commission Information Society and Media is the prevention and management of diseases through research on “Personal Health Systems”. The hallmark of this concept is to empower citizens to adopt an active role in managing their own health status and, in addition, facilitating early diagnosis of diseases”.[26] In this context mobile teledermatology and mobile teledermoscopy has the potential to become an easy applicable tool for everyone and may open the door for a new flexible triage system for detection of skin cancer in general and melanoma in particular. A person concerned about a changing mole or a new mole can capture an image of a given lesion with a cellular phone and send it via multimedia messaging service (MMS) to a specialized telemedicine centre for triage. Certainly, the legal aspects concerning teleconsultations have to be revaluated based on a new definition of doctor-patient-relationship. Moreover, prospective, randomized clinical studies are needed to test and standardize the proposed mobile triage system for pigmented skin lesions. In conclusion, we foresee that in the near future there will be an icon on the screen of cellular phones allowing to seek directly for a telemedical consultation including advice for dermatological conditions and allowing a virtual triage for new and suspicious moles. So, mobile teledermatology and mobile teledermoscopy is paving the way for early melanoma recognition by enhanced self examination in the spirit of the eHealth program of the European Commission for Information Society and Media.
